# Investigation of hydrogen plasma treatment for reducing defects in silicon quantum dot superlattice structure with amorphous silicon carbide matrix

**DOI:** 10.1186/1556-276X-9-72

**Published:** 2014-02-12

**Authors:** Shigeru Yamada, Yasuyoshi Kurokawa, Shinsuke Miyajima, Makoto Konagai

**Affiliations:** 1 Department of Physical Electronics, Tokyo Institute of Technology, Meguro-ku, Tokyo 152-8552, Japan; 2 Photovoltaics Research Center (PVREC), Tokyo Institute of Technology, Meguro-ku, Tokyo 152-8552, Japan

**Keywords:** Silicon quantum dot, Hydrogen plasma treatment, Defect density, Hydrogen diffusion

## Abstract

We investigate the effects of hydrogen plasma treatment (HPT) on the properties of silicon quantum dot superlattice films. Hydrogen introduced in the films efficiently passivates silicon and carbon dangling bonds at a treatment temperature of approximately 400°C. The total dangling bond density decreases from 1.1 × 10^19^ cm^-3^ to 3.7 × 10^17^ cm^-3^, which is comparable to the defect density of typical hydrogenated amorphous silicon carbide films. A damaged layer is found to form on the surface by HPT; this layer can be easily removed by reactive ion etching.

## Background

Solar cells that use nanomaterials have attracted interest for their potential as ultra-high efficiency solar cells [1]. The conversion efficiency limit of a single-junction solar cell strongly depends on the band gap of the absorber layer, which is known as the Shockley-Queisser limit [2]. To overcome the efficiency limit, various types of quantum dot solar cells, such as quantum size effect type, intermediate band type, and multiexciton generation type, have been proposed [3-5]. The quantum size effect type utilizes the phenomenon that the band gap of a material can be tuned by controlling the diameter of quantum dots, including the periodically arranged narrow-gap quantum dots in a wide-gap dielectric matrix. The fabrication of an amorphous silicon dioxide (a-SiO_2_) matrix including size-controlled silicon quantum dots (Si-QDs) was reported by Zacharias et al. [6]. The size-controlled Si-QDs can be formed by annealing a superlattice with silicon-rich silicon oxide layers and stoichiometric silicon oxide layers, which is called a silicon quantum dot superlattice structure (Si-QDSL). Since this report was published, silicon quantum dots embedded in various wide-gap materials, such as amorphous silicon carbide (a-SiC), amorphous silicon nitride (a-Si_3_N_4_), and hybrid matrices, have been reported [4,7-11]. Further, the quantum size effect can be observed from the measurement of photoluminescence spectra or absorption coefficients [12-14]. The Bloch carrier mobility in a Si-QDSL with an a-SiC matrix is higher than that in a Si-QDSL with an a-SiO_2_ or an a-Si_3_N_4_ matrix [15]. The barrier height between a-SiC and Si quantum dots is lower than those of the other two materials, resulting in the easy formation of minibands [16]. Moreover, the crystallization temperature of a-SiC is lower than those of the other materials. Therefore, in this study, we focus on a Si-QDSL with an a-SiC matrix.

High-temperature annealing above 900°C is needed to fabricate a Si-QDSL with an a-SiC matrix. Several problems, such as dehydrogenation, formation of leakage paths by the crystallization of the a-SiC phase, and dopant diffusion in the intrinsic layer of the solar cell structure, occur during high-temperature annealing, resulting in the degradation of solar cell performance. The dehydrogenation problem has been addressed by hydrogen plasma treatment (HPT) [17]. The crystallization of the a-SiC phase can be prevented by incorporating a small amount of oxygen in the a-SiC matrix [16]. Niobium-doped titanium dioxide (TiO_2_:Nb) can be used as a phosphorus (dopant) diffusion barrier layer for the Si-QDSL solar cell [18]. Using these techniques, an efficiency of 0.39% has been achieved in Si-QDSL solar cells fabricated on insulator substrates [19]. Some researchers have reported the electrical properties of silicon quantum dot solar cells [20,21]. However, clear evidence of the contribution from Si-QDs has not yet been reported because of poor device quality. To improve device quality, the collection efficiency of the photogenerated carrier should be improved. For this purpose, further reduction of the defect density in the Si-QDSL layers and improvement of the p/i interface is significantly important.

In this study, the dependence of hydrogen concentration and defect density in Si-QDSL films on the process temperature of HPT was investigated. Diffusion coefficients of hydrogen in Si-QDSLs for several treatment temperatures were estimated by secondary ion mass spectrometry (SIMS). Hydrogen incorporation was also investigated by Raman scattering spectroscopy. In addition, spin densities were measured by electron spin resonance (ESR) spectroscopy, and the optimal temperature was explored. The influence of HPT on the surface of Si-QDSLs was also investigated. The surface morphologies of Si-QDSLs after HPT were measured by atomic force microscopy (AFM), and the thicknesses of the surface damaged layers were estimated by spectroscopic ellipsometry and cross-sectional transmission electron microscopy (TEM). The etching of the surface damaged layer was performed by reactive ion etching (RIE) using a tetrafluoromethane and oxygen (CF_4_ + O_2_) gas mixture.

## Methods

Forty-period hydrogenated amorphous silicon oxycarbide with a silicon-rich composition (a-Si_0.56_C_0.32_O_0.12_:H)/hydrogenated amorphous silicon oxycarbide (a-Si_0.40_C_0.35_O_0.25_:H) superlattice was deposited on quartz substrates using very-high frequency plasma-enhanced chemical vapor deposition. The source gases were silane (SiH_4_), monomethylsilane (MMS), hydrogen (H_2_), and carbon dioxide (CO_2_). The flow rates of MMS, H_2_, and CO_2_ were fixed as 1.7, 47.5, and 0.4 sccm, respectively. SiH_4_ was intermittently flowed during the deposition of silicon-rich layers. Plasma power density, plasma frequency, deposition temperature, deposition pressure, and electrode distance were 13 mW/cm^2^, 60 MHz, 193°C, 20 Pa, and 3 cm, respectively. The thicknesses of silicon-rich layers and stoichiometric layers were 5 and 2 nm, respectively. The films were thermally annealed at 900°C for 30 min under a forming gas (3% H_2_ + 97% N_2_) atmosphere to form Si-QDs. Film thicknesses of post-annealed samples were 250 ± 10 nm.

After annealing, the samples were exposed to hydrogen plasma to terminate dangling bond defects accompanying hydrogen atoms in the Si-QDSL. The flow rate of H_2_, plasma power density, plasma frequency, process pressure, and electrode distance were 200 sccm, 2.60 W/cm^2^, 60 MHz, 600 Pa, and 3 cm, respectively. The treatment temperature was varied from 200°C to 600°C. To evaluate the hydrogen diffusion coefficient in the Si-QDSL, the samples were treated at 300°C for 20 min, 400°C for 10 min, 500°C for 3 min, and 600°C for 1 min. The depth profiles of the hydrogen concentration were measured by SIMS. In the measurements, Ce^+^ ions were used to measure the hydrogen depth profiles. Also, the depth was calibrated by the etching rate of the Si-QDSL. Crystalline silicon was used as the standard sample to evaluate the hydrogen concentration. The accuracy of the hydrogen concentration by the SIMS measurement was ± 40%. In addition, for measurements of Raman scattering spectra and ESR, treatment temperature was varied from 200°C to 600°C and the treatment time was fixed at 60 min.

The thicknesses of surface damaged layers formed by 60-min HPT were estimated by spectroscopic ellipsometry and cross-sectional TEM. The surface morphologies of Si-QDSLs after a 60-min HPT were measured by AFM. The etching of the surface damaged layer was performed by RIE using CF_4_ + O_2_ gas (4% O_2_ + 96% CF_4_). The gas flow rate, process pressure, and plasma power density were 10 sccm, 4 Pa, and 0.221 W/cm^2^, respectively. The surface morphologies after etching were evaluated by AFM and spectroscopic ellipsometry.

## Results and discussion

**Figure 1:**
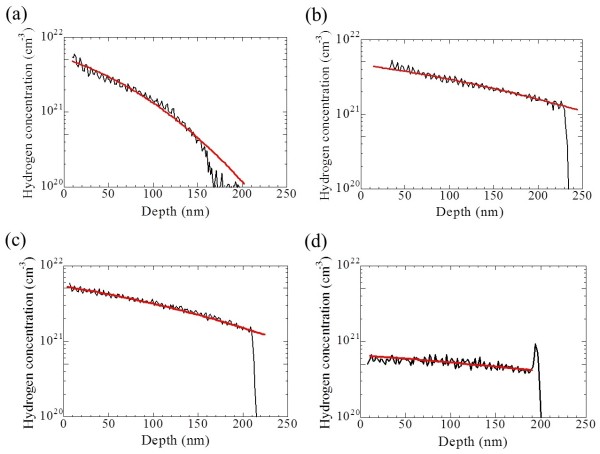
**Depth profiles of hydrogen concentrations. (a)** At 300°C for 20 min. **(b)** At 400°C for 10 min. **(c)** At 500°C for 3 min. **(d)** At 600°C for 1 min.

**Figure 2:**
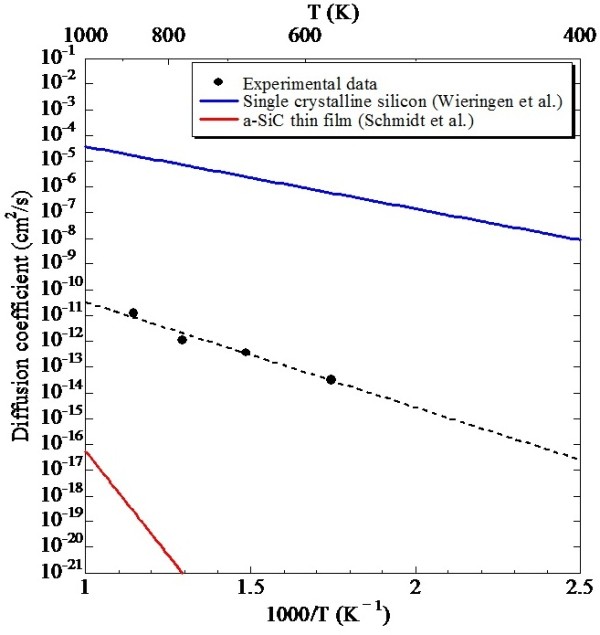
**Arrhenius plot of diffusion coefficient of hydrogen in Si-QDSLs.** The calculated diffusion coefficients of single-crystal silicon by van Wieringen et al. [22] and the estimated diffusion coefficients of an a-SiC thin film with hydrogen concentration of 0.4 ± 0.1 at.% by Schmidt et al. [23] are also described.

An average hydrogen concentration of 8.2 × 10^22^ cm^-3^ was almost uniformly incorporated in the superlattice films before thermal annealing. After annealing at 900°C, the average hydrogen concentration decreased to 1.4 × 10^20^ cm^-3^. After HPT, the hydrogen concentration increased. Figure 1 shows the depth profiles of hydrogen concentrations of Si-QDSL samples treated at 300°C for 20 min, 400°C for 10 min, 500°C for 3 min, and 600°C for 1 min. The oscillations with small amplitudes in the depth profiles are due to the matrix effect caused by carbon in the Si-QDSLs. The influence of the matrix effect can be negligible. In addition, structure of the Si-QDSL is almost uniform in the depth direction. Therefore, one can believe the shape of the hydrogen depth profile, which is important to determine the hydrogen diffusion coefficient. The diffusion coefficients can be estimated from these depth profiles. The hydrogen diffusion process follows the diffusion equation

(1) $$\frac{\partial C}{\partial t}=D\frac{\partial^{2}C}{\partial x^{2}},$$

where *D* is the diffusion coefficient and *C* is the hydrogen concentration at depth *x* and time *t*. If the initial hydrogen concentration at all *x* can be assumed to zero except for *x* = 0, and if the hydrogen concentration at *x* = 0 can be fixed as *C*_0_ for *t* > 0, the following solution is obtained:

(2) $$C\;\left(x,t\right)=C_{0}\mathrm{erfc}\;\left(\frac{x}{2\sqrt{\mathrm{Dt}}}\right),$$

where the function erfc(*z*) is a complementary error function. The average diffusion coefficients were estimated by fitting the depth profiles with Equation 2. Red lines in Figure 1 indicate the fitting curves based on Equation 2. The calculated diffusion coefficients for each temperature were described by dots in Figure 2. The diffusion coefficient obeys Arrhenius law:

(3) $$D=D_{0}\exp\left(-\frac{\mathrm{\Delta E}}{k_{B}T}\right),$$

where *D*_0_ denotes the preexponential factor, Δ*E* is the activation energy, and *k*_B_ is the Boltzmann constant. From the result of the fitting by least squares method, *D*_0_ and Δ*E* were estimated as 3.93 × 10^-7^ cm^2^/s and 0.81 eV, respectively. The calculated diffusion coefficients of single-crystal silicon by van Wieringen et al. [22] and the estimated diffusion coefficients of an a-SiC thin film with hydrogen concentration of 0.4 ± 0.1 at.% by Schmidt et al. [23] are also described in Figure 2. *D*_0_ and Δ*E* for single-crystal silicon and the a-SiC thin film are 9.67 × 10^3^ cm^2^/s and 0.48 eV and 0.71 cm^2^/s and 3.2 eV, respectively. Compared with these Δ*E* values, Δ*E* for Si-QDSL is relatively close to the Δ*E* for single-crystal Si. Such small Δ*E* indicates that the interstitial diffusion in Si-QDs is dominant because the thickness of the a-SiCO layers is too thin to work as barriers against hydrogen diffusion; this is due to the wide band gap and polar bonds of a-SiC [24].

From the depth profiles of Si-QDSLs for a treatment temperature of 600°C, hydrogen concentration was found to drastically decrease. Saturation hydrogen concentration after sufficient treatment was estimated at approximately 1.0 × 10^21^ cm^-3^, indicating that the hydrogen concentration at the surface drastically decreases because the loss of adsorbed hydrogen atoms is dominant at high temperatures.

The defect densities of Si-QDSLs after 60-min HPT for several treatment temperatures were measured by ESR. The defect densities originating from silicon dangling bonds (Si-DBs) and carbon dangling bonds (C-DBs) were also estimated. The waveform separation of the obtained differentiated waves originating from both Si-DBs and C-DBs were so difficult that the ratios between the densities of Si-DBs and C-DBs were estimated by the following equations [25]:

(4) gx,γ=∑n=0121-xgSi¯n+xγgC¯n1-x+xγCn12xn1-x12-n,

(5) $$N_{\mathrm{Si}-\mathrm{DB}}=\frac{1}{1+\gamma }\;N_{\mathrm{Total}-\mathrm{DB}}$$

and

(6) $$N_{C-\mathrm{DB}}=\frac{\gamma }{1+\gamma }N_{\mathrm{Total}‒\mathrm{DB}},$$

where *N*_Total*-*DB_, *N*_Si-DB_, and *N*_C-DB_ are the densities of total dangling bonds (Total-DBs), Si-DBs, and C-DBs, respectively. *y* is the ratio of *N*_C-DB_ to *N*_Si-DB_ and *x* is the composition ratio of C to Si. $\bar{g_{\mathrm{Si}}}\left(n\right)$ is the average *g* value of Si-DB surrounded by (12 - *n*) Si atoms and *n* C atoms, $\bar{g_{C}}\left(n\right)$ is the average *g* value of a C-DB surrounded by (12 - *n*) Si atoms and *n* C atoms, and *g*(*x*, *y*) is the measured *g* value. Although this expression is derived for an a-Si_1-*x*_C_*x*_ alloy system, it is believed to be valid for Si-QDSL with an a-SiC matrix, which can be considered as an approximately homogeneous material, since the dangling bond defect density in Si-QDs is much lower than that of the a-SiC matrix, and the dangling bonds on Si-QD surfaces are passivated by the a-SiC matrix. An average composition ratio of 0.40 was used.

**Figure 3:**
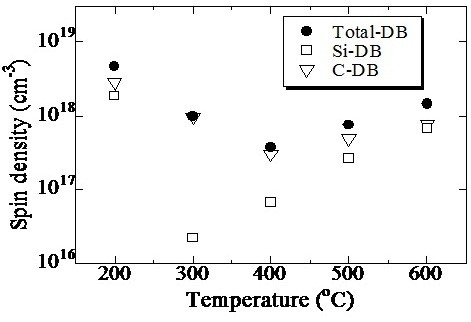
Spin densities of Si-QDSLs after a 60-min HPT.

*N*_Total-DB_, *N*_Si-DB_, and *N*_C-DB_ for several treatment temperatures are shown in Figure 3. Post-HPT, Si-QDSL defect density (1.1 × 10^19^ cm^-3^) clearly reduced compared with the defect density before HPT. The defect density for 200°C treatment is still high because hydrogen diffusion is insufficient. Hydrogen intrusion depth for 60-min HPT can be estimated to be below 100 nm, and a several dangling bonds remain in the deep area of the film. The defect density for 300°C treatment is lower than that at 200°C. A large amount of hydrogen reaches the interface of the film and substrate during the 60-min HPT. The measured *g* value in this sample was 2.00275, which is quite similar to the *g* value of C-DB, meaning that *N*_Si-DB_ is less than *N*_C-DB_. Based on Equation 5, *N*_Si-DB_ is estimated to be 2.2 × 10^16^ cm^-3^, indicating that Si-DBs can be efficiently passivated by the incorporated hydrogen. For the 400°C treatment, defect density decreases to 3.7 × 10^17^ cm^-3^, which is comparable with the defect density of an a-SiC film. The *g* value for 400°C treatment was higher than that for 300°C treatment, indicating that C-DBs - which are dominant in the total-DBs - significantly decrease despite the increment in Si-DBs. For the 500°C treatment, defect density increases despite efficient hydrogen incorporation in the Si-QDSL, showing that the hydrogen atoms are thermally activated from the Si-H bond state to the interstitial state above 300°C and from the C-H bond state to the interstitial state above 400°C. These temperatures mostly correspond to the temperatures of dehydrogenation from Si-H bonds and C-H bonds, which are approximately above 300°C [26] and 450°C to 550°C [27], respectively. In the 500°C treatment sample, a large amount of hydrogen atoms were in the interstitial sites; they did not contribute to the passivation of the dangling bonds.

**Figure 4:**
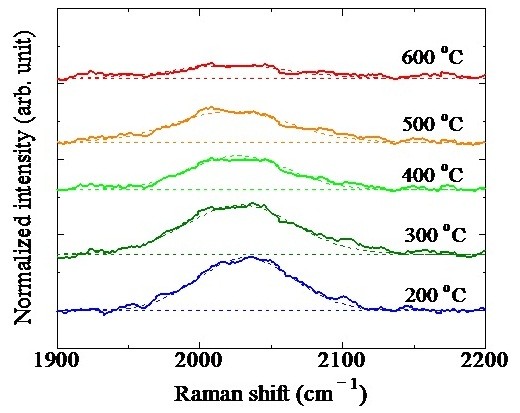
Raman spectra of Si-QDSLs after a 60-min HPT.

Figure 4 shows the Raman spectra of the Si-QDSLs after 60-min HPT at different temperatures. The peak found between 2,000 and 2,100 cm^-1^ corresponds to the Raman shift originating from the stretching mode of Si-H_*n*_ bonds. The intensity of the peak from Si-H_*n*_ bonds gradually weakens as the treatment temperature increases, indicating that the Si-H_*n*_ bonds decomposed by the thermal activation of terminal hydrogen atoms. This trend agrees with the increment of *N*_Si-DB_.

**Figure 5:**
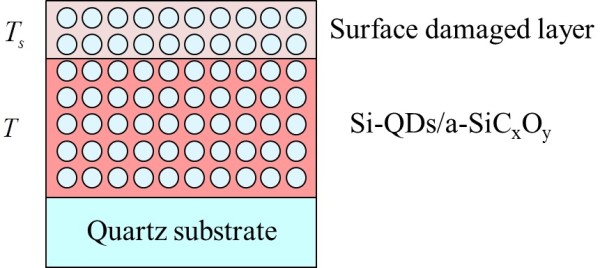
Schematic of the structure of Si-QDSLs after HPT for the parameter fitting of spectroscopic ellipsometry.

During HPT, the surface is damaged by atomic hydrogen. The thickness of the surface damaged layer is dependent on the treatment temperature. The thickness of the surface damaged layer was estimated by spectroscopic ellipsometry. A schematic of the structure used for the analysis is shown in Figure 5. The Tauc-Lorentz model was applied to the optical modeling of the Si-QDSL layer, and the surface damaged layer was assumed to be the effective medium approximation (EMA) layer in which 50% void exists. The estimated thicknesses of the Si-QDSL layers *T*, the thicknesses of the surface damaged layers *T*_s_, and the mean square error (MSE) of each fitting are summarized in Table 1. *T*_*s*_ of an as-annealed Si-QDSL was approximately 2 nm, while the *T*_s_ of the treated Si-QDSLs drastically increased, indicating that the Si-QDSL structure in the surface region was broken by the atomic hydrogen.

**Table 1:** Thicknesses estimated by fitting of the spectroscopic ellipsometry measurements of Si-QDSLs

**Parameters**	**300°C**	**400°C**	**500°C**	**600°C**
MSE	11.56	12.22	13.37	13.30
*T*_s_ (nm)	33.1	11.5	15.2	6.5
*T* (nm)	167.7	212.8	224.7	246.1

**Figure 6:**
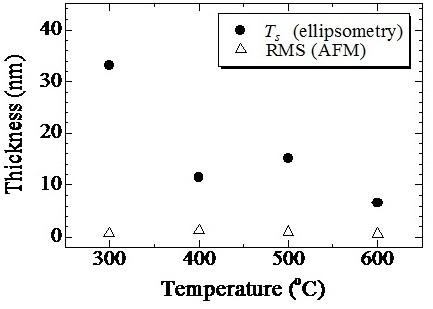
RMS roughness measured by AFM and thicknesses of surface damaged layers estimated by spectroscopic ellipsometry.

**Figure 7:**
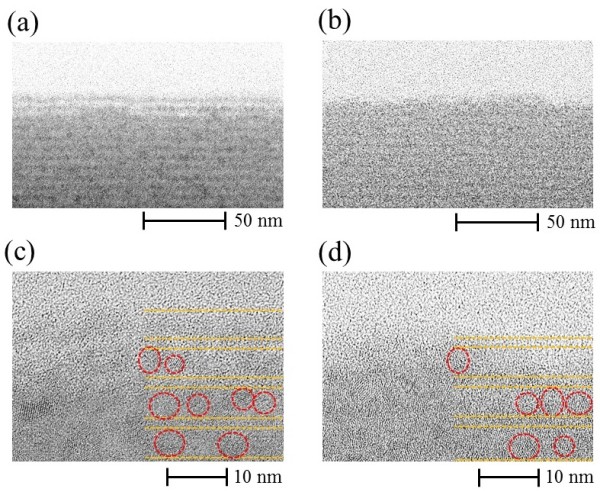
**Cross-sectional TEM images.** At the near-surface of **(a)** 350°C treatment sample, **(b)** 600°C treatment sample, **(c)** magnified image of 350°C treatment sample, and **(d)** magnified image of 600°C treatment sample.

The thicknesses *T* and *T*_s_ strongly depend on the treatment temperature. *T* decreases as the treatment temperature increases; this tendency is related to the hydrogen concentration at the near-surface for each treatment temperature. A large amount of hydrogen introduced into amorphous silicon contributes to the structural reconstruction by breaking the weak Si-Si bonds [28,29]. Further, surface morphologies were measured by AFM. The root mean square (RMS) surface roughness of the samples is shown in Figure 6. RMS surface roughness is almost independent of the treatment temperature, whereas the damaged layer thickness measured by spectroscopic ellipsometry decreased with treatment temperature, indicating that HPT at low temperature introduces a damaged layer with lower refractive index than that of Si-QDSL. To investigate further, TEM observations of the Si-QDSLs were conducted. Figure 7a,b shows TEM images of the 350°C and 600°C treatment samples, and Figure 7c,d shows the magnified images of each sample. In the magnified images, existence of the Si-QDs is indicated using red circles. The irradiated electrons are transmitted through the sample without scattering in the white region, showing that the material density at the near surface is extremely low in the white region. Detailed analysis of the TEM images revealed that the two periods of superlattice layers were completely removed by 350°C HPT. Two or three periods of superlattice layers were found to be damaged. On the other hand, for the 600°C treatment sample, no removal of the layers was observed during the HPT treatment; only the one-period superlattice layer was damaged. This result agrees with the thickness of the damaged layer estimated by the spectroscopic ellipsometry.

**Figure 8:**
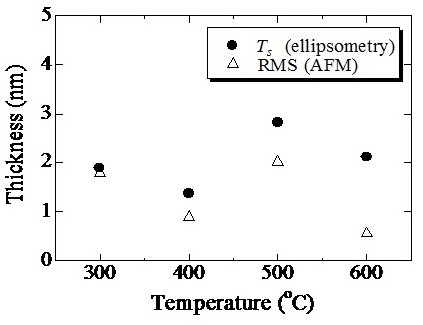
RMS roughness measured by AFM and thicknesses of the surface damaged layers of Si-QDSLs after RIE.

The damaged layer is defective and no longer acts as a Si-QDSL. Therefore, the existence of the damaged layer is a cause of the degradation of Si-QDSL solar cell performance. The removal of the damaged layer without additional damage is very important. Therefore, etching of the damaged layer was performed using RIE. RMS roughness measured by AFM and the damaged layer thicknesses estimated by spectroscopic ellipsometry of the Si-QDSLs after RIE are shown in Figure 8. The estimated thicknesses of the Si-QDSL layers *T*, the thicknesses of the surface damaged layers *T*_s_, and the MSE of each fitting are summarized in Table 2. The observed RMS roughness was less than 3 nm, which was almost the same as that of the sample before RIE. The thicknesses of the surface damaged layers estimated by spectroscopic ellipsometry were almost the same as those of the RMS roughness. In general, surface roughness is also modeled using the EMA model for ellipsometry analysis; thus, the estimated *T*_s_ reflects surface roughness, and no damaged layer exists on the surface. These results clearly indicate that RIE can remove the damaged layer without additional damage to the sample; RIE is therefore the key to improve the film quality of Si-QDSLs and the p/i interface in Si-QDSL solar cells.

**Table 2:** Thicknesses estimated by fitting of the spectroscopic ellipsometry measurements of surface-etched Si-QDSLs

**Parameters**	**300°C**	**400°C**	**500°C**	**600°C**
MSE	14.94	10.80	14.72	15.90
*T*_s_ (nm)	1.9	1.4	2.8	2.1
*T* (nm)	165.0	172.8	171.2	245.5

## Conclusions

Hydrogen plasma treatment temperature dependences of defect densities and hydrogen concentrations in Si-QDSLs as well as the surface morphologies of Si-QDSLs were investigated. Hydrogen could be quickly incorporated as the treatment temperature increases. On the other hand, dehydrogenation of hydrogen atoms terminating the dangling bonds is dominant during high-temperature treatments. The optimal treatment temperature was found to be approximately 400°C, and a defect density of 3.7 × 10^17^ cm^-3^ was achieved, which is comparable to the defect density of a typical a-SiC:H film. In addition, damaged layer was found to form on the surface by HPT; this damaged layer can be easily removed by RIE without additional damage to the sample. Thus, HPT and damaged layer removal process are very important for the fabrication of Si-QDSL solar cells.

## Competing interests

The authors declare that they have no competing interests.

## Authors’ contributions

SY carried out the experiments and the calculations. MK supervised the work and finalized the manuscript. YK and SM participated in the design of the study and helped to draft the manuscript. All authors read and approved the final manuscript.
